# Schilddrüse und SARS-CoV-2

**DOI:** 10.1007/s41969-022-00173-0

**Published:** 2022-09-02

**Authors:** Georg Zettinig

**Affiliations:** 1 Schilddrüsenpraxis Josefstadt, Laudongasse 12/8, 1080 Wien, Österreich; 2 Medizinische Universität Wien, Wien, Österreich

**Keywords:** SARS-CoV‑2, COVID-19, Schilddrüse, Morbus Basedow, Chronische Immunthyreoiditis Hashimoto, SARS-CoV‑2, COVID-19, Thyroid gland, Gravesʼ disease, Chronic autoimmune thyroiditis Hashimoto

## Abstract

Es gibt verschiedene Wechselwirkungen zwischen einer SARS-CoV-2-Infektion und der Schilddrüse, bidirektional in beide Richtungen: Bei einer schweren COVID-19-Infektion sind Veränderungen der Schilddrüsenhormonspiegel ein Marker für eine schlechtere Prognose. SARS-CoV‑2 scheint sowohl direkt mit Thyreozyten zu interagieren als auch das Immunsystem zu modulieren und Immunthyreopathien triggern zu können. Bereits 2020 wurde die „SARS-CoV-2-assoziierte Thyreoiditis“ bei Patienten mit COVID-19 beschrieben, die ähnlich einer subakuten Thyreoiditis verläuft, allerdings typischerweise schmerzlos. Es gibt inzwischen verschiedenste Berichte über das Auftreten einer chronischen Immunthyreoiditis und eines Morbus Basedow sowohl nach Virusinfektion als auch nach Impfung. Eine bestehende Schilddrüsenerkrankung scheint weder mit einem höheren Risiko für eine SARS-CoV-2-Infektion noch mit einem schwereren Krankheitsverlauf assoziiert zu sein. In der vorliegenden Arbeit wird der derzeitige Wissensstand bezüglich Schilddrüse und SARS-CoV‑2 zusammengefasst.

## Abstract (en)

There are several interactions between a SARS-CoV‑2 infection and the thyroid, bidirectionally in both directions. In severe COVID-19 infection, changes in thyroid hormone levels are a marker for poorer prognosis. SARS-CoV‑2 appears to interact directly with thyrocytes as well as modulate the immune system and trigger autoimmune thyroid disease. As early as 2020, SARS-CoV‑2 associated thyroiditis was described in patients with COVID-19, which is similar to subacute thyroiditis but typically painless. There are now a wide variety of reports on the occurrence of chronic autoimmune thyroiditis and Gravesʼ disease following both viral infection and vaccination. Existing thyroid disease does not appear to be associated with either a higher risk of SARS-CoV‑2 infection or a more severe disease course. The present paper summarizes the current knowledge regarding the thyroid gland and SARS-CoV‑2.

## Einleitung

Seit Beginn der Pandemie 2020 kam es fast überall auf der Welt zu einschneidenden Veränderungen in verschiedensten Lebenssituationen, und der Umgang mit SARS-CoV‑2 (Severe acute respiratory syndrome coronavirus 2) prägt nun unseren täglichen Alltag. Im Gegensatz zu den früheren Infektionswellen mit SARS-CoV‑1 (Severe acute respiratory syndrome coronavirus 1) und MERS (Middle East respiratory syndrome-related coronavirus) kam es zu keinem spontanen Sistieren der Infektionsausbrüche, sondern das Virus breitete sich rapide in mehreren Wellen über den gesamten Erdball aus. SARS-CoV‑2 hat eine Wechselwirkung mit verschiedenen Organsystemen, beeinflusst das Gerinnungssystem und führt zu pulmonalen und systemischen Entzündungsreaktionen [1]. Neben einer direkten Schädigung von Zellen führt eine Infektion mit SARS-CoV‑2 auch zu einer indirekten Schädigung des Organismus durch immunvermittelte inflammatorische Reaktionen, bei denen auch Zytokine, das Komplementsystem und die Blutgerinnung involviert zu sein scheinen [2–5].

Wie schon SARS-CoV‑1 infiziert auch SARS-CoV‑2 menschliche Zellen über sein Spike-Protein, das an den Angiotensin-Converting-Enzyme 2(ACE2)-Rezeptor der Wirtszelle [6] bindet. Eine Schlüsselrolle dabei spielt die Transmembran-Protease Serin 2 (TMPRSS2), die die Bindung des Spike-Proteins an den ACE2-Rezeptor aktiviert [7]. Thyreozyten exprimieren sowohl ACE2- als auch TMPRSS2-Rezeptoren in großer Zahl, die Expression beider Rezeptoren ist in Schilddrüsengewebe sogar höher als in der Lunge [8–10]. Eine weitere Rolle bei der Interaktion des Virus mit Thyreozyten scheint die Transmembran-Protease Serin 4 (TMPRSS4) zu spielen, die in der Schilddrüse ebenfalls in hoher Dichte exprimiert wird [11].

Computersimulationen deuten darauf hin, dass die ACE2-Expression in der Schilddrüse mit verschiedenen immunmodulatorischen Prozessen korreliert (CD8+ T‑Zellen, Interferonantwort, B‑Zellen, natürliche Killerzellen) [10]. Neben ACE2- und TMPRSS2-Rezeptoren scheinen noch verschiedene andere Oberflächenstrukturen an den Zellen die Aufnahme des Virus zu modulieren. Eine davon ist Integrin, dessen Expression durch Tetrajodthyronin (T4) reguliert wird [12]. Thyreozyten exprimieren wie verschiedene andere periphere Zellen in großer Zahl olfaktorische Rezeptoren. Die Zerstörung der olfaktorischen Rezeptoren in der Nase ist die Grundlage für die Anosmie bei COVID-19, und es wurde postuliert, dass die Schädigung dieser Rezeptoren auch in anderen Organen wie der Schilddrüse zu Gewebsschädigungen führen kann [13].

In einer Obduktionsserie von Patienten, die an COVID-19 verstarben, wurde in neun von 25 Proben SARS-CoV‑2 im Schilddrüsengewebe nachgewiesen [14].

## Veränderungen der Schilddrüsenhormonspiegel bei hospitalisierten COVID-19-Patienten

Relativ bald nach Beginn der Pandemie erschienen erste Berichte über Veränderungen der Schilddrüsenfunktionsparameter bei hospitalisierten COVID-19-Patienten [15–18]. Die meisten dieser Studien berichteten, dass niedrigere Werte von Thyroidea-stimulierendem Hormon (TSH), freiem Tetrajodthyronin (fT4) und vor allem freiem Trijodthyronin (fT3) mit einer schlechteren Prognose der COVID-19-Erkrankung assoziiert waren [15, 19].

Niedrige TSH-Werte waren auch bei mildem und moderatem Verlauf mit niedrigen Cycle treshold(Ct)-Werten in der Polymerasekettenreaktion (PCR) assoziiert, und es bestand in dieser Patientengruppe ein Zusammenhang zwischen niedrigen T3-Werten mit systemischer Inflammation und Fieber [16, 17, 19].

Kürzlich wurden in einer Metaanalyse 20 Studien zu diesem Thema aufgearbeitet [20]: Bei Patienten mit schweren COVID-19-Verläufen war die Inzidenz abnormer Schilddrüsenblutwerte höher und die Serumspiegel von fT4 (nicht signifikant) und fT3 (signifikant) waren niedriger als bei jenen COVID-19-Patienten, die keinen schweren Verlauf hatten. Die Unterschiede zwischen an COVID-19 Verstorbenen und Überlebenden waren ähnlich. Niedrige fT3- und fT4-Werte sowie niedrige TSH-Werte im Serum sind während des stationären Aufenthalts bei COVID-19-Patienten mit einer erhöhten Mortalität assoziiert. Umgekehrt sind die freien T3-Werte und auch die freien T4- und die TSH-Werte umso niedriger, je schwerer der Verlauf der COVID-19-Erkrankung ist [20].

## Sind bestehende Schilddrüsenerkrankungen ein Risikofaktor für den schweren Verlauf einer COVID-19-Erkrankung?

Als in Österreich im Rahmen des Pandemiemanagements der Begriff des „Risikopatienten“ geprägt wurde, fanden sich viele Schilddrüsenpatientinnen und -patienten darin wieder und es kam zu einer großen Verunsicherung sowohl unter Ärzten als auch Patienten. Zu Beginn der Pandemie war die evidenzbasierte Datenlage noch unübersichtlich, und Patientinnen und Patienten mit Autoimmunerkrankungen der Schilddrüse wurden von verschiedenen Expertengremien als „Risikopatienten“, teilweise sogar als „Hochrisikopatienten“ klassifiziert [21]. Der Impfservice der Gemeinde Wien vergab bei Vorliegen einer chronischen Immunthyreoiditis bevorzugt Impftermine, da im Rahmen der Vormerkungen Patienten mit „einer Autoimmunerkrankung (z. B. Hashimoto)“ als Personen mit sonstigen schweren Erkrankungen in der Gruppe „Hochrisikopatienten“ eingestuft wurden [22]. In den österreichischen „Anwendungsempfehlungen des Nationalen Impfgremiums“ zu COVID-19-Impfungen werden „Vorerkrankungen und Umstände, die ein erhöhtes Risiko für einen schweren Verlauf von COVID-19 bedingen können“ aufgeführt. Auch hier wurden in den verschiedenen Versionen dieser Anwendungsempfehlungen „Autoimmunerkrankungen“ als erhöhtes Risiko für einen schweren COVID-19-Verlauf aufgelistet. Die Wortwahl hinsichtlich Immunthyreoiditis wurde mit zunehmender Evidenz allerdings zusehends abgeschwächt; in der aktuellen Version dieser Empfehlungen lässt die Formulierung „Chronische entzündliche Darmerkrankungen, Autoimmunerkrankungen und rheumatische Erkrankungen“ in erster Linie nicht mehr an Patienten mit einer chronischen Immunthyreoiditis denken [23].

Vorsicht ist geboten, nicht näher definierte Schilddrüsenerkrankungen generell als Risikofaktor bei SARS-CoV-2-Infektionen zu betrachten. Zum Beispiel identifizierte eine kürzlich erschienene Metaanalyse 20 aus 672 publizierten Studien für eine weitere Analyse und berichtete, dass das Vorliegen von „thyroid disorders“ bei Spitalsaufnahme von COVID-19-Patienten mit einer schlechteren Prognose assoziiert sei [24]. Die Autoren spezifizieren allerdings nicht näher, welche Schilddrüsenerkrankungen analysiert wurden, und machen keine näheren Angaben zu verschiedenen relevanten Schilddrüsenerkrankungen wie Knoten oder Autoimmunthyreopathien. Die Literaturzitate der Metaanalyse lassen vermuten, dass „thyroid disorders“ hier die oben beschriebenen niedrigeren peripheren Schilddrüsenhormon- bzw. TSH-Werte meint, die bekannterweise mit einer schlechteren Prognose der COVID-19-Erkrankung assoziiert sind.

Bereits bald nach Beginn der Pandemie gab es erste Hinweise, dass Autoimmunerkrankungen der Schilddrüse einen gewissen protektiven Effekt beim Verlauf der COVID-19-Erkrankung haben könnten. Muller et al. [25] verglichen die Charakteristika von Patienten, die 2019 in der Prä-COVID-19-Ära auf einer Intensivstation betreut wurden, mit jenen, die 2020 nach Beginn der Pandemie intensivmedizinisch betreut wurden: Unter den 2020er-Intensivpatienten war die Prävalenz sowohl an autoimmunen als auch an nicht-autoimmunen Erkrankungen der Schilddrüse niedriger als unter den 2019er-Intensivpatienten. Dies deuten die Autoren als Hinweis, dass weder autoimmune noch nicht-autoimmune Schilddrüsenerkrankungen einen Risikofaktor für einen schweren COVID-19-Verlauf darstellen [25].

Bereits im August 2020 erschien die Untersuchung eines Kollektivs von 3703 SARS-CoV-2-positiven New Yorkern, unter denen sich 251 mit Thyroxintherapie einer Unterfunktion befanden, 22 davon aufgrund einer chronischen Immunthyreoiditis. Die Schilddrüsenhormontherapie war weder mit erhöhtem Risiko für Hospitalisierung noch mit mechanischer Beatmung noch mit Tod assoziiert [26]. Dies wurde in einem großen brasilianischen Kollektiv bestätigt: Patienten mit bekannter Schilddrüsenunterfunktion mussten signifikant seltener beatmet werden, hatten einen kürzeren stationären Aufenthalt und es fand sich ein Trend zu niedriger Mortalität während des stationären Aufenthalts [27]. Eine französische Gruppe folgerte, dass eine Schilddrüsenhormontherapie ein Risikofaktor für COVID-19 sein könnte, da in ihrem Kollektiv 20 von 180 COVID-19-Patienten Schilddrüsenhormon einnahmen, was signifikant häufiger war als in der Kontrollgruppe (23/360) [28]. In einem griechischen Kollektiv zeigte sich hingegen, dass die Mortalität unter COVID-19-Erkrankten, die bereits vor Erkrankungsbeginn unter Schilddrüsenhormontherapie standen, signifikant geringer war als bei jenen ohne Schilddrüsenhormontherapie [29]. In Dänemark wurden alle Patienten, die zwischen Februar und September 2020 auf SARS-CoV‑2 getestet wurden, im Detail analysiert; die Behandlung einer Schilddrüsenfunktionsstörung schien hier nicht mit einem höheren Risiko für eine auf SARS-CoV-2-Infektion einherzugehen [30].

Auch wenn die Situation noch nicht ganz klar ist, scheinen die oben aufgelisteten Daten darauf hinzuweisen, dass eine bestehende Schilddrüsenerkrankung, insbesondere die T4-Behandlung einer Unterfunktion (deren Ursache ja oft eine chronische Immunthyreoiditis ist), mit keinem erhöhten Risiko für eine SARS-CoV-2-Infektion oder einen schweren Verlauf einer COVID-19-Erkrankung verbunden zu sein scheint.

## COVID-19, SARS-CoV-2-Impfung und Thyreoiditis

Bereits im Mai 2020 wurde zum ersten Mal über das Auftreten einer subakuten Thyreoiditis nach SARS-CoV-2-Infektion berichtet [31], zahlreiche weitere Fallberichte und Fallserien folgten. Im Juli 2020 erfolgte die detaillierte Beschreibung einer Subgruppe von acht COVID-19-Patienten mit abnormen Schilddrüsenwerten [25]: Zwei hatten eine chronische Immunthyreoiditis, die restlichen sechs die Charakteristika einer subakuten Thyreoiditis mit passagerer, durch Zellzerfall ausgelösten Überfunktion, die in Folge in eine Unterfunktion überging. Wie bei der klassischen subakuten Thyreoiditis de Quervain zeigte sich das Muster einer fokalen Thyreoiditis in Ultraschall und Szintigrafie. Die Entzündung verlief allerdings schmerzlos. Die Thyreoperoxidase(TPO)-Antikörper waren nicht erhöht. Diese als „SARS-CoV‑2 assoziierte Thyreoiditis“ bezeichnete Form der Schilddrüsenentzündung wurde in Folge immer wieder beschrieben und fand sich nicht nur nach Infektion, sondern auch nach Impfung [32–34].

Aus Österreich gibt es bisher keine Daten zu einem möglichen Anstieg der subakuten Thyreoiditis. Eine erste Analyse zweier großer österreichischer Institutionen zeigte ab März 2020 zwar einen leichten Trend, aber keinen signifikanten Anstieg an Patienten mit subakuter Thyreoiditis [21]. Es ist noch unklar, warum offenbar nicht nur nach Infektion, sondern auch nach Impfung Thyreoiditiden auftreten [35]. Auch ist noch unklar, ob es sich bei diesen Wechselwirkungen primär um eine direkte Schädigung der Thyreozyten durch das Virus handelt oder ob sie durch immunmodulatorische Effekte [36] bedingt sind. Bei den bisherigen Berichten handelt es sich um Fallberichte und Fallserien, ein kausaler Zusammenhang durch Fall-Kontrollstudien wurde bisher noch nicht dokumentiert.

Eine SARS-CoV-2-Infektion scheint auch die Ausbildung von TPO-Antikörpern zu induzieren; in verschiedenen prospektiven Studien wird über einen signifikanten Anstieg der TPO-Antikörper nach COVID-Infektion berichtet, der reversibel zu sein scheint [19, 37, 38]. Eine SARS-CoV-2-Infektion scheint eine bestehende Autoimmunerkrankung der Schilddrüse zu exazerbieren [19]. Bei Patienten mit chronischer Immunthyreoiditis führt eine SARS-CoV-2-mRNA-Impfung zu einer vergleichbaren Immunantwort wie bei Schilddrüsengesunden [39].

## COVID-19, SARS-CoV-2-Impfung und Morbus Basedow

Es gibt inzwischen verschiedene Fallberichte, die nicht nur die Erstmanifestation oder das Rezidiv eines Morbus Basedow, sondern auch das Auftreten einer endokrinen Orbitopathie nach SARS-CoV-2-Infektion dokumentieren [40–45].

Auffällig sind die inzwischen zahlreichen Berichte über einen zeitlichen Zusammenhang zwischen SARS-CoV-2-Impfung und anschließendem Auftreten eines Morbus Basedow. Kurz nach der Erstbeschreibung von zwei Patienten mit Morbus Basedow nach SARS-CoV-2-mRNA-Impfung [46] dokumentierten wir zwei unserer Patienten, bei denen sich ebenfalls dieser zeitliche Zusammenhang fand, und veröffentlichten deren klinische Charakteristika [47]. Viele andere Fallberichte und Fallserien folgten, die das Auftreten eines Morbus Basedow nicht nur nach mRNA-Impfung, sondern auch nach Vektor- und Totimpfung beschrieben [48–52]. Auch nach Impfung wird das Auftreten einer endokrinen Orbitopathie beschrieben, unter anderem zwölf Jahre nach Radiojodtherapie bei einer Patientin mit zuvor jahrelang stabilem Verlauf [53].

In der Comirnaty-Zulassungsstudie wird im Supplementary Appendix in Table 11 ein Patient aus der mRNA-Gruppe aufgelistet, der während der Nachbeobachtungszeit thyreoidektomiert wurde, klassifiziert als „serious adverse effect“. Nähere Angaben zum Grund der Thyreoidektomie liegen nicht vor [54].

Kürzlich erschienene Reviews beleuchten ausführlich die möglichen Mechanismen der Induktion einer Autoimmunerkrankung durch eine SARS-CoV-2-Infektion [37], das Triggern einer Schilddrüsenerkrankung durch eine Infektion sowie die möglichen Mechanismen einer impfinduzierten Schilddrüsenerkrankung [36, 55, 56]. Die SARS-CoV-2-Impfung als einer der Eckpfeiler der Pandemiebekämpfung scheint hier ebenso wie die Virusinfektion in einer Wechselwirkung mit der Schilddrüse zu stehen, deren genaues Ausmaß noch nicht völlig verstanden ist.

Inwieweit es sich bei den beschriebenen zeitlichen Assoziationen zwischen Infektion, Impfung und Schilddrüsenerkrankung um kausale Zusammenhänge handelt, muss noch in weiteren Studien untersucht werden. Momentan ist die vorliegende Evidenz auf diesem Gebiet noch sehr unübersichtlich, und die Zeit wird zeigen, welche kausalen Zusammenhänge hier bestehen und wie wir unseren Patienten in diesen speziellen klinischen Situationen am besten helfen können.

## Fazit für die Praxis

Die Wechselwirkung zwischen SARS-CoV‑2 und der Schilddrüse ist bidirektional und noch nicht vollständig verstanden. Bei schweren COVID-19-Infektionen sind Veränderungen der Schilddrüsenfunktionsparameter ein Marker für eine schlechtere Prognose. Möglicherweise können sowohl eine COVID-19-Erkrankung als auch eine SARS-CoV-2-Impfung eine Thyreoiditis bzw. einen Morbus Basedow triggern – Erkrankungen, die im Vergleich zur potenziell tödlichen COVID-19-Infektion allerdings meist sehr gut zu behandeln sind.
